# Glutaminolysis and the Control of Neural Progenitors in Neocortical Development and Evolution

**DOI:** 10.1177/10738584211069060

**Published:** 2022-01-20

**Authors:** Vasiliki Gkini, Takashi Namba

**Affiliations:** 1 Neuroscience Center, HiLIFE—Helsinki Institute of Life Science, University of Helsinki, Helsinki, Finland

**Keywords:** metabolism, neocortex, development, evolution, neural stem/progenitor cells

## Abstract

Multiple types of neural progenitor cells (NPCs) contribute to the development of the neocortex, a brain region responsible for our higher cognitive abilities. Proliferative capacity of NPCs varies among NPC types, developmental stages, and species. The higher proliferative capacity of NPCs in the developing human neocortex is thought to be a major contributing factor why humans have the most expanded neocortex within primates. Recent studies have shed light on the importance of cell metabolism in the neocortical NPC proliferative capacity. Specifically, glutaminolysis, a metabolic pathway that converts glutamine to glutamate and then to α-ketoglutarate, has been shown to play a critical role in human NPCs, both in apical and basal progenitors. In this review, we summarize our current knowledge of NPC metabolism, focusing especially on glutaminolysis, and discuss the role of NPC metabolism in neocortical development, evolution, and neurodevelopmental disorders, providing a broader perspective on a newly emerging research field.

## Introduction

The mammalian neocortex is a recent evolutionary structure that has been linked to the higher cognitive abilities of humans. Neocortical size and shape vary among mammalian species, even within primates (Herculano-Houzel 2019; Rakic 2009; Zilles and others 2013). During the evolution toward modern human, the human acquired the most expanded and convoluted neocortex compared with other primates (Rakic 2009).

**Figure 1.**
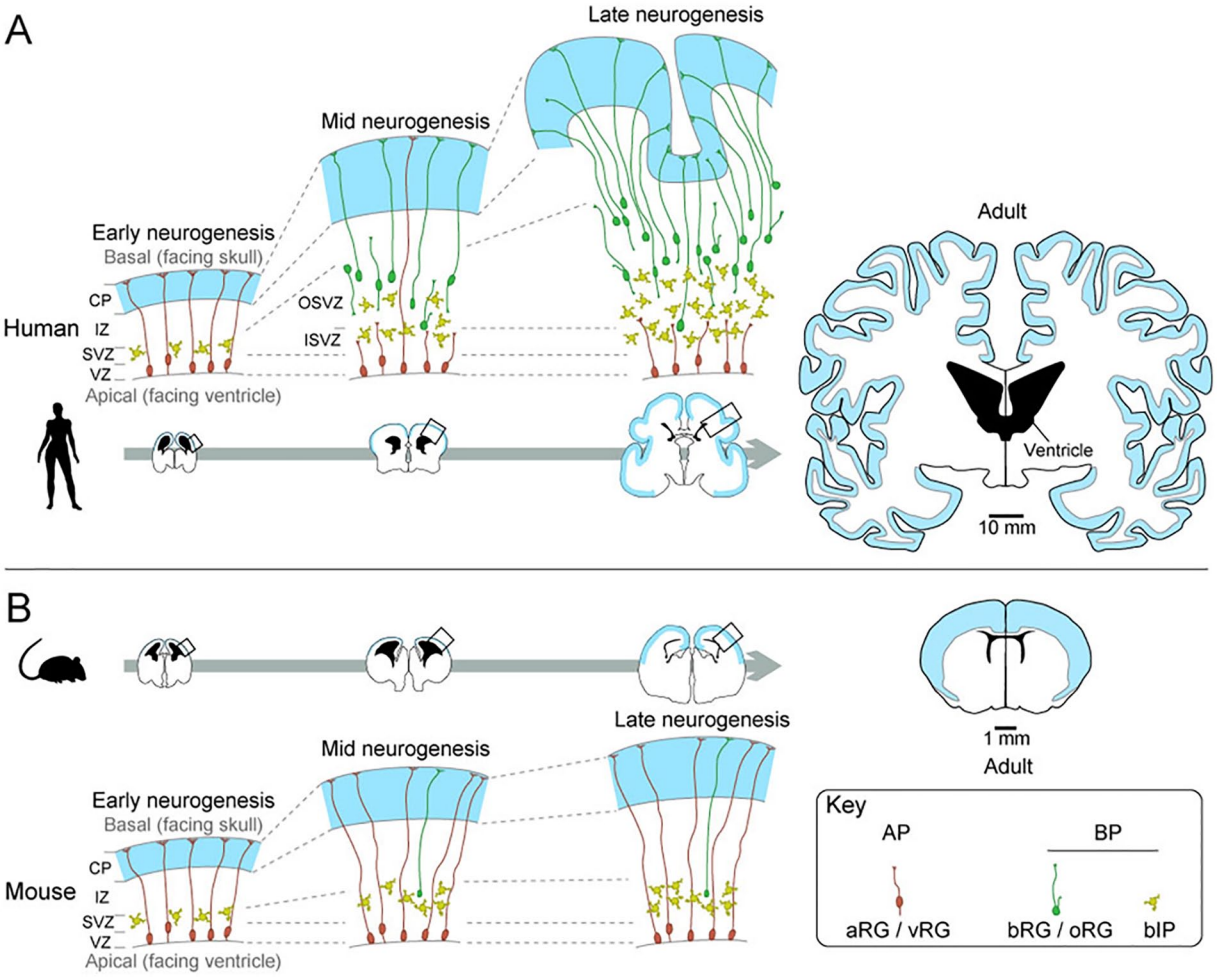
Development of the neocortex in gyrencephalic (human) and lissencephalic (mouse) species. (A) Cartoons of human neocortex at early (9 weeks post conception [wpc], left), mid (15 wpc, middle), and late (22 wpc, right) neurogenesis, which are enlarged representative images of the boxed areas in the coronal brain sections (bottom). The right most coronal section is adult brain. (B) Cartoons of mouse neocortex at early (embryonic day [E] 12.5, left), mid (E14.5, middle), and late (E16.5, right) neurogenesis, which are enlarged representative images of the boxed areas in the coronal brain sections (top). The apical progenitors (APs), mainly consisting of apical radial glia (aRG/vRG), are located in the ventricular zone (VZ), and produce basal progenitors (BPs) in the subventricular zone (SVZ). In the human neocortex at mid to late neurogenesis, both basal intermediate progenitors (bIP) and basal/outer radial glia (bRG/oRG) are multiplied to expand the SVZ into two distinct layers, inner and outer SVZ (ISVZ and OSVZ, respectively). In contrast, the expansion of BP in mouse neocortex is smaller, and there are very few bRGs. The right most coronal sections are adult brains. Blue and black areas in the coronal sections indicate the gray matter and the lateral ventricle, respectively. Scale bars of 10 mm and 1 mm apply to human and mouse coronal brain sections, respectively.

Neocortical expansion depends on the proliferative capacity of neural stem and progenitor cells (NPCs), and the subsequent neuron production (Cárdenas and Borrell 2020; LaMonica and others 2012; Namba and Huttner 2017; Rash and others 2019; Sun and Hevner 2014; Fig. 1). NPCs can be divided into two major classes, apical progenitors (APs), which mainly consist of apical radial glia (aRG, also known as ventricular radial glia, vRG), and basal progenitors (BPs) comprising basal intermediate progenitors (bIPs) and basal radial glia (bRG, also known as outer radial glia, oRG). APs and BPs are located in the ventricular (VZ) and subventricular zone (SVZ) of the developing neocortex, respectively. aRG mainly expand their number during early development of the neocortex, and then start producing BPs at mid to late developmental stages (Cárdenas and Borrell 2020; Namba and Huttner 2017; Sun and Hevner 2014). Since only a small part of aRG directly produce neurons and the rest of them give rise to BPs, which divide and finally differentiate into neurons, the BPs are thought to be the main progenitor cells responsible for neuron production. In species with a larger neocortex, such as human and nonhuman primates, there is an abundance of bRG and bIPs during development, both of which are known to possess high proliferative capacity in these species (Cárdenas and Borrell 2020; LaMonica and others 2012; Namba and Huttner 2017; Sun and Hevner 2014). The SVZ of those species is enlarged to accommodate the expanded BP pool and further subdivided into the inner and outer SVZ (ISVZ and OSVZ, respectively; Smart 2002). In contrast, the developing neocortex of species with a small neocortex, such as mouse, generally contains a smaller number of bRG and few proliferative bIPs (Cárdenas and Borrell 2020; LaMonica and others 2012; Namba and Huttner 2017; Sun and Hevner 2014), making the SVZ relatively thin. Therefore, the expansion of the bRG and bIPs, due to their high proliferative capacity, is the main driving force of the evolutionary expansion of the neocortex (Fig. 1).

Understanding the mechanisms of the BP expansion in human evolution is important for neuroscientists studying not only physiological brain development but also brain development in pathological conditions such as microcephaly and megalencephaly (Juric-Sekhar and Hevner 2019). Recent advances in comparative genomics and transcriptomics allow us to identify genes that are potentially responsible for the BP expansion. The translated proteins of those genes are known to regulate extra cellular signal sensing (e.g., HTR2A, PDGFRB, NOTCH2NL), cell morphology (PALMD), cell cycle progression (e.g., MTOR, TMEM14B), and gene expression (e.g., HOPX, SOX9, TBC1D3, YAP; Andrews and others 2020; Florio and others 2018; Güven and others 2020; Ju and others 2016; Kalebic and others 2019; Kostic and others 2019; Liu and others 2017; Lui and others 2014; Nowakowski and others 2017; Suzuki and others 2018; Vaid and others 2018; Xing and others 2020).

Another cellular process, cell metabolism, has recently been identified as a regulator of human neocortical BP expansion (Namba and others 2020). Human BPs expressing a human-specific mitochondrial protein ARHGAP11B utilize glutaminolysis, a metabolic pathway that converts glutamine to α-ketoglutarate (αKG) via glutamate, to expand their number. Interestingly, another recent study showed that glutaminolysis is also regulated by a microcephaly-associated gene *MCPH1* (Journiac and others 2020), suggesting that an impairment of glutaminolysis could lead to microcephaly. These findings prompt us to review and discuss how glutaminolysis regulates human BP expansion, and thereby the evolutionary enlargement of the human neocortex. This review does not cover morphological dynamics of mitochondria, which is another important aspect of NPC metabolism. Readers interested in this topic may kindly refer to recent seminal review articles (Iwata and Vanderhaeghen 2021; Khacho and others 2019).

## Cell Metabolism in Neural Stem and Progenitor Cells: An Overview

**Figure 2.**
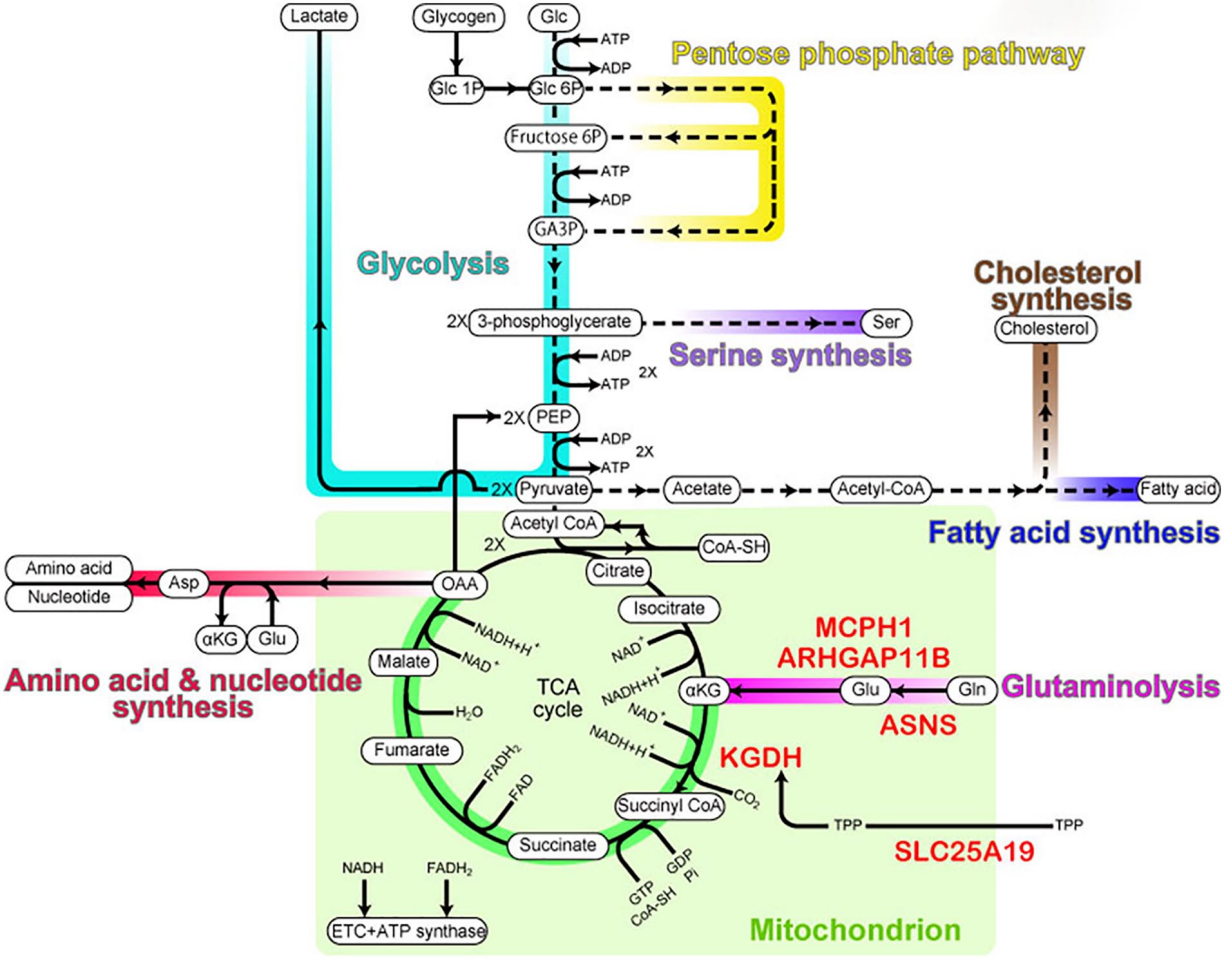
Metabolic pathways in NPCs. Metabolic pathways relevant to the NPC proliferation are color-coded as follows: turquoise, glycolysis; yellow, pentose phosphate pathway; purple, serine synthesis; blue, fatty acid synthesis; brown, cholesterol synthesis; red, amino acid and nucleotide synthesis; magenta, glutaminolysis; green, the three-quarter TCA cycle. The mitochondrial compartment is indicated in light green. αKG, α-ketoglutarate; CoA-SH, coenzyme A; Fructose 6P, fructose 6-phosphate; GA3P, glyceraldehyde 3-phosphate; Glc, glucose; Glc 1P, glucose 1-phosphate; Glc 6P, glucose 6-phosphate; OAA, oxaloacetate; PEP, phosphoenolpyruvic acid; TPP, thiamine pyrophosphate. Proteins important for glutaminolysis are indicated in red.

Most of our knowledge regarding metabolic pathways in NPCs has been obtained from studies using mice. It has been shown that different NPC types exhibit distinct metabolic activities. To provide an overview of the metabolic pathways in NPCs, we shall start from an extensively studied metabolic pathway, glycolysis, to the tricarboxylic acid (TCA) cycle, which supplies oxidative phosphorylation (OXPHOS) with metabolites to produce ATP (Fig. 2).

### Glycolysis

The most well studied and widely utilized energy source for cells is glucose. Glucose is taken up into cells through glucose transporters and then catabolized through the process called glycolysis. The first step of glycolysis is glucose phosphorylation by hexokinase. This phosphorylation produces glucose 6-phosphate, which is processed further in the glycolytic pathway.

As a result of glycolysis, one glucose is finally converted into two pyruvates. In this process, cells consume two ATP to produce four ATP. Pyruvate is then transported to the mitochondria where it is converted to acetyl-CoA, which fuels the TCA cycle. Under anaerobic conditions, pyruvate is converted into lactate. Pyruvate to lactate conversion can also happen in rapidly dividing cells, like cancer cells, under aerobic conditions (referred to as the Warburg effect; Lunt and van der Heiden 2011).

Mouse neocortical aRG show higher glycolytic lactate production compared with bIPs (Khacho and others 2016). The relatively high glycolytic lactate production in aRG is presumably due to a hypoxic environment of the VZ (Komabayashi-Suzuki and others 2019; Lange and others 2016b). Since the onset of angiogenesis in the VZ is slightly later than the onset of aRG expansion, the aRG initially undergo symmetric proliferative division in a hypoxic environment (Komabayashi-Suzuki and others 2019; Lange and others 2016b). Interestingly, even after the onset of angiogenesis, the VZ has lower blood vessel density compared with the SVZ, suggesting that the lower oxygen availability primes aRG to exhibit higher glycolytic lactate production throughout neocortical development. Sustained expression of hypoxia inducible factor 1α (HIF-1α), a transcription factor stabilized by hypoxia, in the aRG throughout corticogenesis supports this notion (Komabayashi-Suzuki and others 2019; Lange and others 2016a). While the cell soma of aRG is always located in the VZ, the basal process spans from the VZ to the pia. Therefore, the oxygen availability is likely different between the cell soma and the basal process, suggesting that aRG might have distinct glycolytic activities within one cell.

A pathological condition has been described, in which reduced angiogenesis could potentially lead to thinning of the cortices and microcephaly-associated disorders. Specifically, deficiency of MFSD7c, an endothelial transporter of the brain blood vessels, results in decreased growth of blood vessels in the VZ and SVZ. In addition, mouse embryos lacking this protein exhibited severe hypoxia and neuronal cell death, potentially leading to reduced brain growth and microcephaly-associated disorders (Kalailingam and others 2020; Meyer and others 2010).

Glucose 6-phosphate is a crossroad of different metabolic pathways. In addition to glucose, glucose 6-phosphate is also provided by a catabolic pathway called glycogenolysis. This pathway is particularly interesting because aRG are known to be enriched in glycogen (Gressens and Evrard 1993). Furthermore, glucose 6-phosphate is diverted to the pentose phosphate pathway (PPP), which consists of the oxidative and the nonoxidative phase, and produces glyceraldehyde 3-phosphate and fructose 6-phosphate while generating NADPH. Silencing of transketolase, a key enzyme of the nonoxidative branch of PPP, reduces cell proliferation of hippocampal NPCs (Zhao and others 2009) and tumor cells (Xu and others 2016), suggesting that PPP might be important for neocortical NPCs as well.

### TCA Cycle

Pyruvate, a final product of glycolysis, is transported into the mitochondria by mitochondrial pyruvate carriers (MPC1 and MPC2). Pyruvate is then converted into acetyl-CoA by pyruvate dehydrogenase. Acetyl-CoA transfers its own two carbons to oxaloacetate (OAA), which originates from pyruvate or amino acids, to form citrate (Fig. 2). Citrate is then converted into α-ketoglutarate (αKG) via isocitrate. The conversion of αKG to succinyl-CoA by α-ketoglutarate dehydrogenase (KGDH) complex is an important step in the TCA cycle for brain development since this is where the interchange of the TCA cycle and glutaminolysis occurs (see below). Succinyl-CoA is then converted into OAA through multiple intermediate metabolites, that is, succinate, fumarate, and malate (Fig. 2).

While the major role of the TCA cycle is to provide NADH and FADH_2_ for OXPHOS (see below), each intermediate metabolite of the TCA cycle is also important for anabolic pathways (Berg and others 2002). For example, citrate is used for the synthesis of lipids and αKG is used to produce nonessential amino acids. In addition, pathways starting from oxaloacetate can produce glucose (gluconeogenesis), amino acids, and nucleotides. To sustain the efflux of these TCA intermediates (cataplerosis), the cells must replenish them with a similar influx (anaplerosis). An important anaplerotic source is the amino acid glutamine and its metabolism, a process called glutaminolysis (DeBerardinis and others 2008; Lunt and van der Heiden 2011).

### Oxidative Phosphorylation

Oxidative phosphorylation (OXPHOS), also known as the electron transport chain, is the main source of energy under aerobic conditions. During OXPHOS, NADH and FADH_2_ from the TCA cycle transfer electrons to O_2_ while generating ATP (Berg and others 2002). In principle, one NADH and one FADH_2_ generate three and two ATP, respectively; thus, one glucose can provide the OXPHOS with NADH and FADH_2_, through glycolysis and the TCA cycle, to generate 34 ATP. The other possible source of NADH and FADH_2_ is beta oxidation of fatty acids (Cooper 2000). It has been shown that OXPHOS activity gradually increases along with neuronal differentiation (Khacho and others 2016). In the mouse embryonic neocortex, bIPs exhibit higher OXPHOS activity than aRG (Khacho and others 2016). Since impairment of Cpt1 and trimethyllysine dioxygenase (TMLHE), key molecules of the beta oxidation of fatty acids, mainly affects aRG abundance (Bankaitis and Xie 2019; Knobloch and others 2017; Xie and others 2016), bIPs may utilize NADH and FADH_2_, which are produced by the TCA cycle.

As discussed above, the higher OXPHOS activity of the bIPs might be a response to the relatively higher oxygen availability from the blood in the SVZ (Komabayashi-Suzuki and others 2019; Lange and others 2016a). These differences are also observed in the adult mouse hippocampus (Knobloch and Jessberger 2017). Quiescent neural stem cells show lower OXPHOS and exhibit higher glycolysis than the intermediate progenitor cells in the adult mouse hippocampus, suggesting that the changes in OXPHOS and glycolysis activity along with neuronal differentiation are commonly observed in NPCs at various developmental stages. In contrast to NPCs in the embryonic neocortex, these two types of hippocampal NPCs are localized in the same germinal zone, namely, subgranular zone, thus their oxygen availability should not be different. It is interesting to understand alternative mechanisms, other than oxygen availability, which regulate the balance of OXPHOS versus glycolysis in NPCs.

Other important molecules produced by OXPHOS are the peroxides superoxide and hydrogen peroxide, known as reactive oxygen species (ROS). In a classical view, ROS are cytotoxic molecules causing oxidative stress, for example, DNA damage and oxidation of protein and lipids (Sies and Jones 2020). Recent studies, however, revealed a new role of ROS as signal molecules (Bigarella and others 2014). In mouse embryonic neocortex, mitochondrial ROS production is higher in bIPs relative to aRG (Khacho and others 2016). The mitochondrial ROS production in bIP induces the expression of genes that lead to neuronal differentiation (Khacho and others 2016), suggesting that ROS produced in the mitochondria are exported out of the mitochondria to exert their function. Indeed, inhibition of ROS leakage from mitochondria, so called ROS flash, promotes NPC proliferation (Hou and others 2012).

Taken together, NPCs need to maintain a balance of glycolysis, TCA cycle, and OXPHOS activity, in order to produce enough ATP and other metabolites for cell proliferation, while keeping moderate ROS levels. Because higher lactate-producing glycolysis can suppress the activity of the TCA cycle, this metabolic balance might require an additional fuel source, that is, glutaminolysis.

## Glutaminolysis in Neocortical Development, Evolution, and Disorders

### Glutaminolysis in Neocortical Development and Evolution

Glutaminolysis is the process by which glutamine is converted to glutamate and then to αKG (Yang and others 2017; Fig. 2). Recent studies have shown that glutaminolysis is crucial to neocortical development (Journiac and others 2020; Molinari and others 2009; Namba and others 2020), and interestingly its activity varies among species. Glutaminolysis is present both in mouse and human aRG (Journiac and others 2020), whereas only human BPs, not mouse BPs, exhibit glutaminolysis (Namba and others 2020). Pharmacological inhibition of glutaminolysis in both human fetal BPs and mouse early aRG reduces their proliferative capacity, showing that glutaminolysis is required for NPC expansion (Journiac and others 2020; Namba and others 2020). In conclusion, glutaminolysis contributes to two different events: aRG expansion at an early stage of neocortical development and BP expansion at mid to late stages. However, in mice, glutaminolysis acts only in the aRG expansion. Therefore, the evolutionary expansion of BPs can be partly attributed to glutaminolysis. In this section, we use the term BP to indicate both bIP and bRG, since it remains to be addressed which BP subtypes exhibit glutaminolysis in the human fetal neocortex. However, we can infer that at least bRG might exhibit promoted glutaminolysis since aRG and bRG share gene/protein expression profiles in the human fetal neocortex, including ARHGAP11B and MCPH1, as described below (Florio and others 2015; Journiac and others 2020; Namba and others 2020).

**Figure 3.**
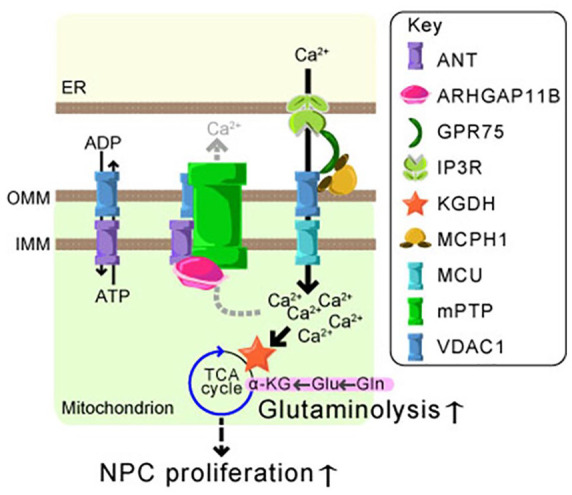
Mitochondrial calcium ion regulation and glutaminolysis in NPCs. Endoplasmic reticulum (ER)-mitochondrial coupling mediated by IP3R, GPR75, VDAC1, and MCPH1 induces Ca^2+^ influx to mitochondria through MCU. The mitochondrial permeability transition pore (mPTP) works with VDAC1 and ANT for Ca^2+^ efflux from mitochondria. ARHGAP11B inhibits mPTP through an interaction with ANT. As a consequence, the mitochondrial Ca^2+^ concentration is increased, thereby KGDH is activated. The activated KGDH consumes α-ketoglutarate (αKG) and, in turn, induces glutaminolysis, which finally leads to NPC expansion. Outer and inner mitochondrial membrane (OMM and IMM, respectively).

What mechanisms drive glutaminolysis in NPCs? Here we will introduce two independent, yet mechanistically interconnected pathways controlling glutaminolysis in NPCs: pathways involving ARHGAP11B and MCPH1/Mcph1. ARHGAP11B is a mitochondrial matrix protein that is encoded by the human-specific gene *ARHGAP11B* (Florio and others 2015; Namba and others 2020) and regulates BP expansion (reviewed in Namba and Huttner 2017). During human evolution, an ancestral (evolutionarily conserved) gene *ARHGAP11A* underwent partial gene duplication followed by a mutation in a splice donor site (Florio and others 2016). The partial gene duplication, which happened just after the lineage split between human and chimpanzee (around 5 million years ago), resulted in an ancestral isoform of *ARHGAP11B* (Florio and others 2016). The molecular function of the ancestral ARHGAP11B appears to be similar to ARHGAP11A. It exhibits nuclear localization, a GTPase-activating protein (GAP) activity, and no effect on BP expansion (Florio and others 2016). Thereafter, the splice donor site mutation occurred on the ancestral *ARHGAP11B*, and this mutation generated modern *ARHGAP11B*, which lost the molecular features of ARHGAP11A (Florio and others 2016), while exhibiting novel subcellular localization and functions (Namba and others 2020). While both ARHGAP11A and ancestral ARHGAP11B are localized in nuclei, modern ARHGAP11B is found in mitochondria (Namba and others 2020). In mitochondria, ARHGAP11B binds to adenine nucleotide translocators (ANTs). ARHGAP11B does not affect the ADP-ATP exchanging ability of ANT; however, it inhibits opening or assembly of the mitochondrial permeability transition pore (mPTP), which regulates mitochondrial Ca^2+^ concentration by releasing Ca^2+^ from mitochondria to cytosol (Doczi and others 2016; Halestrap and Richardson 2015). Its inhibition by ARHGAP11B increases mitochondrial Ca^2+^ concentration (Fig. 3). Ca^2+^ is a known regulator of various enzymes driving the TCA cycle (Denton and others 1972; Williams and others 2015). Of those enzymes, the KGDH complex activity in NPCs, but not in neurons, is known to be increased by mitochondrial Ca^2+^ concentration due to distinct splicing isoforms (Zheng and others 2016). Therefore, an increase in mitochondrial Ca^2+^ concentration by ARHGAP11B might activate the KGDH complex. This in turn accelerates αKG consumption and, consequently, induces compensatory αKG production through glutaminolysis (Fig. 3).

The other pathway controlling glutaminolysis in NPCs is regulated by MCPH1/Mcph1, a microcephaly-associated protein (Journiac and others 2020). MCPH1/Mcph1 is expressed both in aRG and bRG in human fetal neocortex, but only in aRG in mouse embryonic neocortex (Journiac and others 2020). In the early neurogenic period, aRG produce a limited number of neurons but mainly expand their number by symmetric division (Namba and Huttner 2017). A recent study has shown that glutaminolysis in early aRG in mouse neocortex depends on Mcph1. While Mcph1 was originally identified to be linked to the centrosome during cell cycle (Gruber and others 2011; Thornton and Woods 2009), it has been shown to be localized at mitochondria as well (Journiac and others 2020). Mcph1 binds to voltage-dependent anion-selective channel 1 (VDAC1), a mitochondrial outer membrane protein, and G-protein coupled receptor 75 (GPR75), which bridges VDAC1 to inositol-triphosphate receptor (IP3R) on endoplasmic reticulum (ER; Fig. 3). Since Mcph1 interacts with both VDAC1 and GPR75, it might facilitate the indirect interaction between VDAC1 and IP3R, thereby strengthening ER-mitochondria coupling. The ER-mitochondrial coupling allows Ca^2+^ influx from the ER into mitochondria (Szabadkai and others 2006); therefore, Mcph1-expressing mitochondria exhibit higher Ca^2+^ concentration. As described above, elevated mitochondrial Ca^2+^ concentration might lead to an activation of glutaminolysis. Interestingly, VDAC1 is a known component of mPTP (Karch and Molkentin 2014). If ARHGAP11B could inhibit an assembly of mPTP, the presence of ARHGAP11B might increase the proportion of VDAC1 acting on the ER-mitochondrial coupling rather than participating in mPTP.

These two proteins, ARHGAP11B and MCPH1, control mitochondrial Ca^2+^ concentration, and thereby glutaminolysis, through two distinct mechanisms: inhibiting Ca^2+^ release from mitochondria and promoting Ca^2+^ influx into mitochondria (Fig. 3). During the normal neocortical development, these two mechanisms might cooperate to maximize their effects on mitochondrial Ca^2+^ concentration. Compromising their cooperation might potentially lead to abnormal development of neocortex (see below). These results suggest that mitochondrial Ca^2+^ concentration is a key regulator of NPC proliferation. Notably, increased mitochondrial Ca^2+^ concentration in cancer cells promotes cell proliferation when mPTP is inhibited (Marchi and others 2019); thus, this mechanism seems to be shared among highly proliferating cells.

How does glutaminolysis regulate cell proliferation? The final product of glutaminolysis, αKG, is utilized by multiple cellular processes including the TCA cycle (Abla and others 2020; Maus and Peters 2017; Yang and others 2017). Cells exhibiting higher glutaminolysis fuel the TCA cycle with αKG, which in turn could produce more ATP. Concurrently, the TCA cycle could become less reliant on the acetyl-CoA supply from glycolysis. As a consequence, the cycle may stop at oxaloacetate, which is a starting metabolite for nucleotide and amino acid synthesis, as well as gluconeogenesis. This incomplete TCA cycle, which we have named the ¾ TCA cycle (Namba and others 2021), allows glycolysis to produce critical metabolites for various anabolic pathways rather than fueling the TCA cycle. One example is 3-phosphoglycerate, an intermediate metabolite of glycolysis important for serine synthesis. Mutations in enzymes regulating serine synthesis from 3-phosphoglycerate are known to cause microcephaly (Acuna-Hidalgo and others 2014). Given that microcephaly is mainly attributed to an impairment of NPC proliferation, the serine synthesis from 3-phosphoglycerate might regulate NPC proliferative capacity.

Besides the TCA cycle, αKG is involved in amino acid biosynthesis, as well as lipid, especially fatty acid, metabolism. Fatty acid synthesis is particularly important for NPC proliferation since imbalanced fatty acid synthesis, triggered by a fatty acid synthase (FASN) whose mutant is associated with intellectual disability (Najmabadi and others 2011), reduced aRG proliferation in human cerebral organoids (Bowers and others 2020). Furthermore, a regulator of intracellular fatty acid trafficking, fatty acid binding protein 7 (FABP7; also known as brain lipid binding protein, BLBP; Kagawa and others 2019), has been shown to be crucial for both neuroepithelial cells/early aRG proliferation and their morphology (Arai and others 2005). Interestingly, both aRG and bRG, but not bIP, express FABP7. Since the elongated morphology of aRG and bRG is known to be a prerequisite of their higher proliferative capacity (Kalebic and Huttner 2020; Kalebic and Namba 2021), fatty acid metabolism might regulate aRG morphology which in turn influences their proliferative capacity.

Moreover, it acts as a co-substrate for the αKG-dependent dioxygenases, which are responsible for epigenetic regulations (Abla and others 2020; Herr and Hausinger 2018; Islam and others 2018). The role of αKG in epigenetics is well studied in tumorigenesis. Several mutations have been identified in isocitrate dehydrogenases (IDH), which are encoded by the *IDH1* and *IDH2* genes (Pirozzi and Yan 2021). The wild type IDH catalyzes isocitrate to αKG in the TCA cycle. In contrast, the oncogenic mutations endow IDH to possess a novel function, that is, mediating αKG to D-2-hydroxyglutarate (D-2HG) conversion. Because of the structural similarity between αKG and D-2HG, D-2HG inhibits αKG-dependent dioxygenases, such as histone demethylases, which demethylate histones, and Ten-eleven translocation (TET) enzymes, which convert DNA methylation from 5-methyl-cytosine to 5-hydroxy-methylcytosine. As a consequence of the inhibition, cells exhibited an altered epigenetic status, which may inhibit proper cell differentiation (Pirozzi and Yan 2021). Conversely, increased cellular αKG level is known to induce differentiation of human pluripotent stem cells by altering histone methylation (TeSlaa and others 2016). These studies suggest that glutaminolysis regulates the balance of cell proliferation versus differentiation through αKG production followed by epigenetic modification. Since epigenetic regulation is known to control NPC proliferation versus differentiation as well (Albert and others 2017; Hirabayashi and Gotoh 2010), it is particularly interesting to study the relationship between glutaminolysis and epigenetics, and its role in the evolutionary expansion of human fetal BPs.

The intermediate metabolite of glutaminolysis, glutamate, plays an important role in cell proliferation through glutathione synthesis (Lisowski and others 2018). Glutathione is a tripeptide consisting of glutamate, cysteine, and glycine, and exists in reduced and oxidized states that are important for exhibiting its antioxidative activity. Glutathione is synthesized in the cytosol and then transported into the mitochondria. Glutathione neutralizes ROS by oxidizing itself to prevent excessive oxidation of mitochondrial proteins, lipids, and DNA. In addition, as we have discussed above, transient efflux of ROS from mitochondria promotes NPC differentiation (Hou and others 2012). Therefore, neutralization of ROS by glutathione can maintain the stemness of NPCs.

### Where Does Glutamine Come From?

As we discussed above, glutamine appears to be an important metabolite for NPC proliferative capacity, but from where do the NPCs obtain glutamine? We propose three possible sources: cerebrospinal fluid (CSF), blood, and astrocytes. CSF fills the lateral ventricle and is known to be enriched in various metabolites (Wishart and others 2008). The apical membrane of aRG faces the lateral ventricle, and therefore can take various metabolites from the CSF. Since the onset of angiogenesis in the VZ is later than the period when aRG exhibit glutaminolysis, the major source of glutamine in the VZ might be the CSF.

The second possibility is blood. While glutamine in blood does not cross the blood-brain barrier (BBB) in adult brain, glutamine can permeate the brain parenchyma of early fetus because the BBB is still immature (Goasdoué and others 2017). Therefore, at the time when aRG are expanding their pool, they are possibly utilizing glutamine from blood. However, the structure of the human BBB becomes indistinguishable from that of the adult one by 16 weeks post conception (wpc; Goasdoué and others 2017). Therefore, by the time the OSVZ is fully established, the BBB is fully matured and does not allow glutamine to cross the barrier.

The third possible source is astrocytes. In the adult brain, astrocytes take in various metabolites (e.g., glucose), which cross the BBB and convert them to metabolites for other brain cells (García-Cáceres and others 2019). In contrast to the mouse neocortex, in which astrogliogenesis occurs only during the perinatal to neonatal period and overlaps with neurogenesis very little, human astrogliogenesis starts around 16 wpc and overlaps with neurogenesis until the neurogenesis ends around 28 wpc (van den Ameele and others 2014). As there are many astrocytes observed in the OSVZ (Yang and others 2021), they may supply glutamine for BPs. The importance of astrocytes in neocortical expansion has been suggested in the macaque monkey (Rash and others 2019).

### Glutaminolysis in Neurodevelopmental Disorders Associated with Abnormal Brain Size

Studying the etiology of neurodevelopmental disorders is a powerful way to understand the relevance of glutaminolysis to human neocortical development. Of several neurodevelopmental disorders, primary (congenital) microcephaly and megalencephaly are the ones caused by impaired or excessive NPC expansion, respectively (Juric-Sekhar and Hevner 2019; Mirzaa and others 2004; Pinson and others 2019; Verloes and others 1993; Zhang and others 2020). Since both malformations affect higher cognitive functions, developing brain with proper size, not too small nor too large, is important. Here we focus on microcephaly and megalencephaly-associated neurodevelopmental disorders, which are linked directly to glutaminolysis.

Mutations in asparagine synthetase (ASNS), which converts glutamine to glutamate along with the conversion of aspartate to asparagine, cause microcephaly in human (Ruzzo and others 2013). The mutations (A6E and F362V) lead to destabilization of the protein, and thus the protein expression is significantly reduced, suggesting that loss of ASNS protein is the cause of microcephaly. This finding is corroborated by an *Asns* knockout mouse, which exhibits smaller brain size (Ruzzo and others 2013).

Since MCPH1/Mcph1 has been shown to control mitochondrial Ca^2+^ concentration, and thereby glutaminolysis (Journiac and others 2020), dysregulation of mitochondrial Ca^2+^ homeostasis might contribute to the etiology of microcephaly. Extracellular Ca^2+^ enters the mitochondrial intermembrane space through VDAC1 in the outer membrane, and then it moves into the matrix through a Ca^2+^ channel in the inner membrane called mitochondrial Ca^2+^ uniporter (MCU; Fig. 3). Dysregulation of MCU activity caused by mutations in mitochondrial calcium uptake 1 (MICU1), a regulatory component of the channel, leads to abnormal brain development including microcephaly (Logan and others 2014).

Mitochondrial Ca^2+^ concentration regulates the KGDH complex activity. The KGDH complex needs a coenzyme, thiamine pyrophosphate (TPP), to exhibit its activity. Mutations in a gene encoding the mitochondrial TPP carrier, *SLC25A19*, have been identified as a cause of microcephaly (Bottega and others 2019; Kelley and others 2002; Rosenberg and others 2002); thus, the KGDH activity may be important for the proliferative capacity of NPCs.

Another type of cortical malformation associated with brain size is megalencephaly, which shows overgrowth of brain. Genetic mutations affecting the mTOR signaling pathway in the developing brain have been linked to megalencephaly (Barker and Houlston 2003; Poduri and others 2012; Vanhaesebroeck and others 2012). Since mTOR signaling is known to be regulated by cell metabolism and vice versa (Mossmann and others 2018), there might be an involvement of cell metabolism in the etiology of megalencephaly. Interestingly, it has been shown that glutaminolysis activates mTOR signaling in mammalian cells (Durán and others 2012), suggesting that increased activation of glutaminolysis can lead to over-expansion of NPCs, which causes a pathologically bigger brain.

## Perspectives

### Comparative Analysis of Neural Stem/Progenitor Cell Metabolism

Heretofore our view about NPC metabolism in evolution has been obtained mainly by comparing two evolutionary fairly distant species, human versus mouse. To draw a roadmap of the evolutionary metabolic adaptation in NPCs, we need to compare nonhuman primates with human. To this end, brain organoids would be the best practical option (Benito-Kwiecinski and others 2021), though there are some potential disadvantages to consider. (1) A previous study suggests that human brain organoids exhibit increased glycolysis compared to the human fetal neocortex (Bhaduri and others 2020; Pollen and others 2019). This metabolic shift might hinder the understanding of the physiological NPC metabolism. (2) Cells located inside the organoids can only utilize metabolites produced by their surrounding cells and those that permeate from the culture medium to the parenchyma.

To overcome this problem, several studies have tried to vascularize brain organoids (Cakir and others 2019; Daviaud and others 2018; Mansour and others 2018; Shi and others 2020). Interestingly, these vascularized brain organoids are integrated into the host circulatory system upon xenotransplantation to postnatal mouse brain (Daviaud and others 2018; Mansour and others 2018; Shi and others 2020). Alternatively, the vascularized brain organoids can be cultured in a millifluidic chip (Homan and others 2019; Nashimoto and others 2017), which may provide a “pseudocirculation” to the vasculature system. Another way to increase availability of metabolites and oxygen to the cells located inside the organoids is sliced organoid methods (Giandomenico and others 2019; Qian and others 2020). These methodological improvements will enable us to analyze the NPC metabolism in multiple species in a physiologically relevant environment.

### Neural Stem and Progenitor Cell Metabolism beyond Brain

Recent studies shed light on the role of cell metabolism in the evolutionary expansion of human neocortex. Since cell metabolism largely depends on metabolites and oxygen availability (so-called metabolic microenvironment), it is essential to study NPC metabolism in the context of cell-to-cell and maternal-fetal communications.

Owing to the development of BBB, neocortical NPCs, especially BPs, are unable to take essential metabolites directly from the blood (García-Cáceres and others 2019). The metabolism of NPCs largely relies on a metabolite supply from endothelial cells and astrocytes (García-Cáceres and others 2019). In addition to these cells, there is a possibility that other cell types in the OSVZ, such as neurons and BPs themselves, compose the metabolic microenvironment. It is well known that highly proliferating tumor cells exhibit a high level of glycolysis-derived lactate production, and the lactate is transported out to extracellular spaces to form a microenvironment that favors tumor growth (Parks and others 2020).

Metabolite availability in the fetal blood is controlled by the maternal fetal communication happening in the placenta. The placenta forms the placenta barrier, which controls the exchange of molecules between maternal blood and fetal blood. Fetal circulation, a characteristic unique to fetuses, consists of blood from the placenta that bypasses the liver and flows directly to the brain, meaning that metabolite availability in fetal blood is mainly determined by the placenta (Bowman and others 2021). Thus, the maternal metabolic status significantly influences fetal metabolism, and thereby can affect fetal NPC behavior. Indeed, neocortical development in mouse embryos is known to be influenced by maternal factors (Stepien and others 2020). It has been shown that maternal diabetes induced by high-fat diet leads to abnormal expansion of NPCs and reduction of their neuronal differentiation in the mouse embryonic neocortex (Rash and others 2018), suggesting that maternal diet is a factor that can influence fetal NPC proliferation and neocortical development.

**Figure 4.**
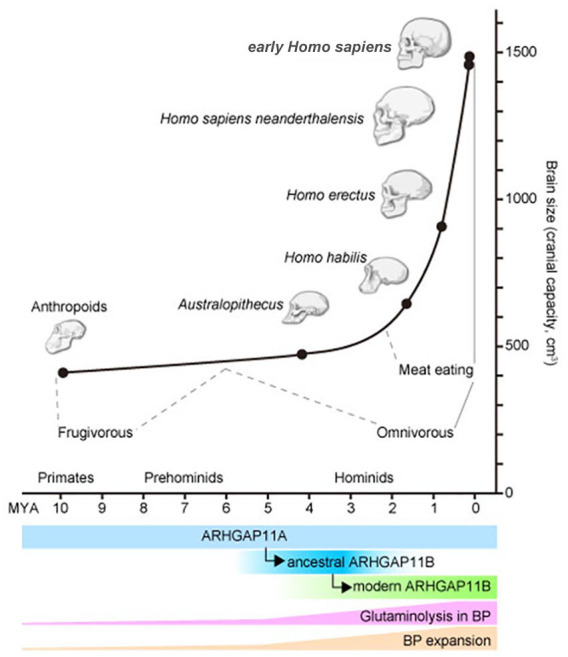
Summary of human evolution, glutaminolysis, BP expansion, and ARHGAP11B. Top: Summary of evolutionary changes in brain size (ordinate; cranial capacity in cm^3^) and time (abscissa; million years ago [MYA]). Different human ancestors at different time points are indicated as black circles with representative images of the skull. Their dietary preferences with strong evidence (solid gray lines) and inference (dashed gray lines) are indicated. Bottom: Timing of ancestral (blue) and modern (green) ARHGAP11B generation from ARHGAP11A (light blue). Presumed activity of glutaminolysis in BPs (pink) and BP expansion (light orange) relative to these in anthropoids are depicted as a height of the crossbars.

During human evolution, dietary availability acted as a strong selective pressure (Andrews and Johnson 2020; Cunnane and Crawford 2014; Luca and others 2010). Ancestral apes are thought to have been as frugivorous as present-day apes, such as chimpanzees (Andrews and Johnson 2020; Cunnane and Crawford 2014; Luca and others 2010). Due to constant climate changes, that happened around 5–3 million years ago (MYA), the availability of fruits in the habitat of early hominins (Ardipithecus and Australopithecus) was gradually decreased, and as a consequence, the early hominins adapted to eat a harder and more fibrous diet, such as roots (Andrews and Johnson 2020; Cunnane and Crawford 2014; Luca and others 2010). Thereafter around 2 MYA, early *Homo*, such as *Homo habilis*, became omnivorous, including meat, and could utilize tools and fire to process food to be more digestible (Andrews and Johnson 2020; Cunnane and Crawford 2014; Luca and others 2010; Fig. 4). When we take the changes in brain size into consideration, the brain size expansion happened only in early *Homo*, who presumably also ate meat, but not in the early hominins, suggesting a relationship between meat diet and brain size expansion. Interestingly, this timeline appears to coincide with the evolution of the ARHGAP11B gene: ancestral ARHGAP11B, which does not stimulate the BP expansion, was generated around 5 MYA, and the modern ARHGAP11B arose sometime between 5–0.5 MYA (Florio and others 2016; Fig. 4). Meat, a rich source of protein and fat, can influence the maternal nutriture, and thereby the metabolite supply to the fetal brain. In turn, this may have allowed BPs to take advantage of an evolutionary adapted metabolism, involving glutaminolysis, at highest efficiency.

Studying NPC metabolism will help us understand how cells, tissues, organisms, and environment interact with each other to develop our brain, and ultimately, what makes us human.

## References

[bibr1-10738584211069060] Abla H Sollazzo M Gasparre G Iommarini L Porcelli AM . 2020. The multifaceted contribution of α-ketoglutarate to tumor progression: an opportunity to exploit? Semin Cell Dev Biol 98:26–33. 31175937 10.1016/j.semcdb.2019.05.031

[bibr2-10738584211069060] Acuna-Hidalgo R Schanze D Kariminejad A Nordgren A Kariminejad MH Conner P , and others. 2014. Neu-laxova syndrome is a heterogeneous metabolic disorder caused by defects in enzymes of the l-serine biosynthesis pathway. Am J Hum Genet 95:285–93. 10.1016/j.ajhg.2014.07.012 PMC4157144 25152457

[bibr3-10738584211069060] Albert M Kalebic N Florio M Lakshmanaperumal N Haffner C Brandl H , and others. 2017. Epigenome profiling and editing of neocortical progenitor cells during development. EMBO J 36:2642–58. 10.15252/embj.201796764 PMC5579386 28765163

[bibr4-10738584211069060] Andrews MG Subramanian L Kriegstein AR . 2020. Mtor signaling regulates the morphology and migration of outer radial glia in developing human cortex. eLife 9:1–21. 10.7554/eLife.58737 PMC7467727 32876565

[bibr5-10738584211069060] Andrews P Johnson RJ . 2020. Evolutionary basis for the human diet: consequences for human health. J Intern Med 287:226–37. 10.1111/joim.13011 31733113

[bibr6-10738584211069060] Arai Y Funatsu N Numayama-Tsuruta K Nomura T Nakamura S Osumi N. 2005. Role of Fabp7, a downstream gene of Pax6, in the maintenance of neuroepithelial cells during early embryonic development of the rat cortex. J Neurosci 25:9752–61. 10.1523/JNEUROSCI.2512-05.2005 PMC6725737 16237179

[bibr7-10738584211069060] Bankaitis VA Xie Z. 2019. The neural stem cell/carnitine malnutrition hypothesis: new prospects for effective reduction of autism risk? J Biol Chem 294:19424–35. 10.1074/jbc.AW119.008137 PMC6916470 31699893

[bibr8-10738584211069060] Barker KT Houlston RS . 2003. Overgrowth syndromes: is dysfunctional PI3-kinase signalling a unifying mechanism? Eur J Hum Genet 11:665–70. 10.1038/sj.ejhg.5201026 12939652

[bibr9-10738584211069060] Benito-Kwiecinski S Giandomenico SL Sutcliffe M Riis ES Freire-Pritchett P Kelava I , and others. 2021. An early cell shape transition drives evolutionary expansion of the human forebrain. Cell 184:2084–2102.e19. 33765444 10.1016/j.cell.2021.02.050 PMC8054913

[bibr10-10738584211069060] Berg J Tymoczko J Stryer L. 2002. Biochemistry. 5th ed. WH Freeman.

[bibr11-10738584211069060] Bhaduri A Andrews MG Mancia Leon W Jung Diane Shin D Allen D , and others. 2020. Cell stress in cortical organoids impairs molecular subtype specification. Nature 578:142–8. 10.1038/s41586-020-1962-0 PMC7433012 31996853

[bibr12-10738584211069060] Bigarella CL Liang R Ghaffari S . 2014. Stem cells and the impact of ROS signaling. Development (Cambridge) 141:4206–18. 10.1242/dev.107086 PMC4302918 25371358

[bibr13-10738584211069060] Bottega R Perrone MD Vecchiato K Taddio A Sabui S Pecile V , and others. 2019. Functional analysis of the third identified SLC25A19 mutation causative for the thiamine metabolism dysfunction syndrome 4. J Hum Genet 64:1075–81. 10.1038/s10038-019-0666-5 PMC6886476 31506564

[bibr14-10738584211069060] Bowers M Liang T Gonzalez-Bohorquez D Kempermann G Fö C Correspondence SJ , and others. 2020. FASN-dependent lipid metabolism links neurogenic stem/progenitor cell activity to learning and memory deficits. Stem Cell 27:1–12. 10.1016/j.stem.2020.04.002 32386572

[bibr15-10738584211069060] Bowman CE Arany Z Wolfgang MJ . 2021. Regulation of maternal–fetal metabolic communication. Cell Mol Life Sci 78:1455–86. 10.1007/s00018-020-03674-w PMC7904600 33084944

[bibr16-10738584211069060] Cakir B Xiang Y Tanaka Y Kural MH Parent M Kang YJ , and others. 2019. Engineering of human brain organoids with a functional vascular-like system. Nat Methods 16:1169–75. 10.1038/s41592-019-0586-5 PMC6918722 31591580

[bibr17-10738584211069060] Cárdenas A Borrell V. 2020. Molecular and cellular evolution of corticogenesis in amniotes. Cell Mol Life Sci 77:1435–60. 10.1007/s00018-019-03315-x PMC11104948 31563997

[bibr18-10738584211069060] Cooper GM . 2000. The mechanism of oxidative phosphorylation. Sinauer Associates.

[bibr19-10738584211069060] Cunnane SC Crawford MA . 2014. Energetic and nutritional constraints on infant brain development: implications for brain expansion during human evolution. J Hum Evol 77:88–98. 24928072 10.1016/j.jhevol.2014.05.001

[bibr20-10738584211069060] Daviaud N Friedel RH Zou H. 2018. Vascularization and engraftment of transplanted human cerebral organoids in mouse cortex. eNeuro 5(6):ENEURO.0219-18.2018. 10.1523/ENEURO.0219-18.2018 PMC6243198 30460331

[bibr21-10738584211069060] DeBerardinis RJ Lum JJ Hatzivassiliou G Thompson CB . 2008. The biology of cancer: metabolic reprogramming fuels cell growth and proliferation. Cell Metab 7:11–20. 18177721 10.1016/j.cmet.2007.10.002

[bibr22-10738584211069060] Denton RM Randle PJ Martin BR . 1972. Stimulation by calcium ions of pyruvate dehydrogenase phosphate phosphatase. Biochem J 128:161–3. 10.1042/bj1280161 PMC1173580 4343661

[bibr23-10738584211069060] Doczi J Torocsik B Echaniz-Laguna A de Camaret BM Starkov A Starkova N , and others. 2016. Alterations in voltage-sensing of the mitochondrial permeability transition pore in ANT1-deficient cells. Sci Rep 6:26700. 27221760 10.1038/srep26700 PMC4879635

[bibr24-10738584211069060] Durán RV Oppliger W Robitaille AM Heiserich L Skendaj R Gottlieb E , and others. 2012. Glutaminolysis activates Rag-mTORC1 signaling. Mol Cell 47:349–58. 10.1016/j.molcel.2012.05.043 22749528

[bibr25-10738584211069060] Florio M Albert M Taverna E Namba T Brandl H Lewitus E , and others. 2015. Human-specific gene ARHGAP11B promotes basal progenitor amplification and neocortex expansion. Science 347:1465–70. 10.1126/science.aaa1975 25721503

[bibr26-10738584211069060] Florio M Heide M Pinson A Brandl H Albert M Winkler S , and others. 2018. Evolution and cell-type specificity of human-specific genes preferentially expressed in progenitors of fetal neocortex. eLife 7:e32332. 29561261 10.7554/eLife.32332 PMC5898914

[bibr27-10738584211069060] Florio M Namba T Paabo S Hiller M Huttner WB . 2016. A single splice site mutation in human-specific ARHGAP11B causes basal progenitor amplification. Sci Adv 2:e1601941. 27957544 10.1126/sciadv.1601941 PMC5142801

[bibr28-10738584211069060] García-Cáceres C Balland E Prevot V Luquet S Woods SC Koch M , and others. 2019. Role of astrocytes, microglia, and tanycytes in brain control of systemic metabolism. Nat Neurosci 22:7–14. 30531847 10.1038/s41593-018-0286-y

[bibr29-10738584211069060] Giandomenico SL Mierau SB Gibbons GM Wenger LMD Masullo L Sit T , and others. 2019. Cerebral organoids at the air–liquid interface generate diverse nerve tracts with functional output. Nat Neurosci 22:669–79. 10.1038/s41593-019-0350-2 PMC6436729 30886407

[bibr30-10738584211069060] Goasdoué K Miller SM Colditz PB Björkman ST . 2017. Review: The blood-brain barrier; protecting the developing fetal brain. Placenta 54:111–6. 10.1016/j.placenta.2016.12.005 27939102

[bibr31-10738584211069060] Gressens P Evrard P . 1993. The glial fascicle: an ontogenic and phylogenic unit guiding, supplying and distributing mammalian cortical neurons. Dev Brain Res 76:272–7. 10.1016/0165-3806(93)90218-y 8149596

[bibr32-10738584211069060] Gruber R Zhou Z Sukchev M Joerss T Frappart PO Wang ZQ . 2011. MCPH1 regulates the neuroprogenitor division mode by coupling the centrosomal cycle with mitotic entry through the Chk1–Cdc25 pathway. Nat Cell Biol 13:1325–34. 10.1038/ncb2342 21947081

[bibr33-10738584211069060] Güven A Kalebic N Long KR Florio M Vaid S Brandl H , and others. 2020. Extracellular matrix-inducing Sox9 promotes both basal progenitor proliferation and gliogenesis in developing neocortex. eLife 9:e49808. 32191207 10.7554/eLife.49808 PMC7105383

[bibr34-10738584211069060] Halestrap AP Richardson AP . 2015. The mitochondrial permeability transition: a current perspective on its identity and role in ischaemia/reperfusion injury. J Mol Cell Cardiol 78:129–41. 10.1016/j.yjmcc.2014.08.018 25179911

[bibr35-10738584211069060] Herculano-Houzel S . 2019. Life history changes accompany increased numbers of cortical neurons: a new framework for understanding human brain evolution. Progr Brain Res 250:179–216. 10.1016/bs.pbr.2019.06.001 31703901

[bibr36-10738584211069060] Herr CQ Hausinger RP . 2018. Amazing diversity in biochemical roles of Fe(II)/2-oxoglutarate oxygenases. Trends Biochem Sci 43:517–32. 10.1016/j.tibs.2018.04.002 PMC6014900 29709390

[bibr37-10738584211069060] Hirabayashi Y Gotoh Y . 2010. Epigenetic control of neural precursor cell fate during development. Nat Rev Neurosci 11:377–88. 10.1038/nrn2810 20485363

[bibr38-10738584211069060] Homan KA Gupta N Kroll KT Kolesky DB Skylar-Scott M Miyoshi T , and others. 2019. Flow-enhanced vascularization and maturation of kidney organoids in vitro. Nat Methods 16:255–62. 10.1038/s41592-019-0325-y PMC6488032 30742039

[bibr39-10738584211069060] Hou Y Ouyang X Wan R Cheng H Mattson MP Cheng A . 2012. Mitochondrial superoxide production negatively regulates neural progenitor proliferation and cerebral cortical development. Stem Cells 30:2535–47. 10.1002/stem.1213 PMC3479374 22949407

[bibr40-10738584211069060] Islam MS Leissing TM Chowdhury R Hopkinson RJ Schofield CJ . 2018. 2-Oxoglutarate-dependent oxygenases. Annu Rev Biochem 87:585–620. 29494239 10.1146/annurev-biochem-061516-044724

[bibr41-10738584211069060] Iwata R Vanderhaeghen P . 2021. Regulatory roles of mitochondria and metabolism in neurogenesis. Curr Opin Neurobiol 69:231–40. 10.1016/j.conb.2021.05.003 PMC8415079 34171617

[bibr42-10738584211069060] Journiac N Gilabert-Juan J Cipriani S Benit P Liu X Jacquier S , and others. 2020. Cell metabolic alterations due to Mcph1 mutation in microcephaly. Cell Rep 31:107506. 32294449 10.1016/j.celrep.2020.03.070

[bibr43-10738584211069060] Ju XC Hou QQ Sheng AL Wu KY Zhou Y Jin Y , and others. 2016. The hominoid-specific gene TBC1D3 promotes generation of basal neural progenitors and induces cortical folding in mice. eLife 5:e18197. 27504805 10.7554/eLife.18197 PMC5028191

[bibr44-10738584211069060] Juric-Sekhar G Hevner RF . 2019. Malformations of cerebral cortex development: molecules and mechanisms. Annu Rev Pathol Mech Dis 14:293–318. 10.1146/annurev-pathmechdis-012418-012927 PMC6938687 30677308

[bibr45-10738584211069060] Kagawa Y Umaru BA Ariful I Shil SK Miyazaki H Yamamoto Y , and others. 2019. Role of FABP7 in tumor cell signaling. Adv Biol Regul 71:206–18. 10.1016/j.jbior.2018.09.006 30245263

[bibr46-10738584211069060] Kalailingam P Wang KQ Toh XR Nguyen TQ Chandrakanthan M Hasan Z , and others. 2020. Deficiency of MFSD7c results in microcephaly-associated vasculopathy in Fowler syndrome. J Clin Investig 130:4081. 32369449 10.1172/JCI136727 PMC7410059

[bibr47-10738584211069060] Kalebic N Gilardi C Stepien B Wilsch-Bräuninger M Long KR Namba T , and others. 2019. Neocortical expansion due to increased proliferation of basal progenitors is linked to changes in their morphology. Cell Stem Cell 24:535–550.e9. 30905618 10.1016/j.stem.2019.02.017

[bibr48-10738584211069060] Kalebic N Huttner WB . 2020. Basal progenitor morphology and neocortex evolution. Trends Neurosci 43:843–53. 10.1016/j.tins.2020.07.009 32828546

[bibr49-10738584211069060] Kalebic N Namba T . 2021. Inheritance and flexibility of cell polarity: a clue for understanding human brain development and evolution. Development (Cambridge) 148:dev199417. 10.1242/dev.199417 PMC8451944 34499710

[bibr50-10738584211069060] Karch J Molkentin JD . 2014. Identifying the components of the elusive mitochondrial permeability transition pore. Proc Natl Acad Sci U S A 111:10396–7. 10.1073/pnas.1410104111 PMC4115577 25002521

[bibr51-10738584211069060] Kelley RI Robinson D Puffenberger EG Strauss KA Holmes Morton D . 2002. Amish lethal microcephaly: a new metabolic disorder with severe congenital microcephaly and 2-ketoglutaric aciduria. Am J Med Genet 112:318–26. 10.1002/ajmg.10529 12376931

[bibr52-10738584211069060] Khacho M Clark A Svoboda DS Azzi J MacLaurin JG Meghaizel C , and others. 2016. Mitochondrial dynamics impacts stem cell identity and fate decisions by regulating a nuclear transcriptional program. Cell Stem Cell 19:232–47. 10.1016/j.stem.2016.04.015 27237737

[bibr53-10738584211069060] Khacho M Harris R Slack RS . 2019. Mitochondria as central regulators of neural stem cell fate and cognitive function. Nat Rev Neurosci 20:34–48. 30464208 10.1038/s41583-018-0091-3

[bibr54-10738584211069060] Knobloch M Jessberger S. 2017. Metabolism and neurogenesis. Cur Opin Neurobiol 42:45–52. 10.1016/j.conb.2016.11.006 27915086

[bibr55-10738584211069060] Knobloch M Pilz GA Ghesquière B Kovacs WJ Wegleiter T Moore DL , and others. 2017. A fatty acid oxidation-dependent metabolic shift regulates adult neural stem cell activity. Cell Rep 20:2144–55. 10.1016/j.celrep.2017.08.029 PMC5583518 28854364

[bibr56-10738584211069060] Komabayashi-Suzuki M Yamanishi E Watanabe C Okamura M Tabata H Iwai R , and others. 2019. Spatiotemporally dependent vascularization is differently utilized among neural progenitor subtypes during neocortical development. Cell Rep 29:1113–1129.e5. 31665628 10.1016/j.celrep.2019.09.048

[bibr57-10738584211069060] Kostic M Paridaen JTML Long KR Kalebic N Langen B Grübling N , and others. 2019. YAP activity is necessary and sufficient for basal progenitor abundance and proliferation in the developing neocortex. Cell Rep 27:1103–1118.e6. 31018127 10.1016/j.celrep.2019.03.091 PMC6486488

[bibr58-10738584211069060] LaMonica BE Lui JH Wang X Kriegstein AR . 2012. OSVZ progenitors in the human cortex: an updated perspective on neurodevelopmental disease. Curr Opin Neurobiol 22:747–53. 10.1016/j.conb.2012.03.006 PMC3402619 22487088

[bibr59-10738584211069060] Lange C Garcia MT Decimo I Bifari F Eelen G Quaegebeur A , and others. 2016a. Relief of hypoxia by angiogenesis promotes neural stem cell differentiation by targeting glycolysis. EMBO J 35:924–41. 10.15252/embj.201592372 PMC5207321 26856890

[bibr60-10738584211069060] Lange C Turrero Garcia M Decimo I Bifari F Eelen G Quaegebeur A , and others. 2016b. Relief of hypoxia by angiogenesis promotes neural stem cell differentiation by targeting glycolysis. EMBO J 35:924–41. 10.15252/embj.201592372 PMC5207321 26856890

[bibr61-10738584211069060] Lisowski P Kannan P Mlody B Prigione A . 2018. Mitochondria and the dynamic control of stem cell homeostasis. EMBO Rep 19:e45432. 29661859 10.15252/embr.201745432 PMC5934764

[bibr62-10738584211069060] Liu J Liu W Yang L Wu Q Zhang H Fang A , and others. 2017. The primate-specific gene TMEM14B marks outer radial glia cells and promotes cortical expansion and folding. Cell Stem Cell 21:635–649.e8. 29033352 10.1016/j.stem.2017.08.013

[bibr63-10738584211069060] Logan CV Szabadkai G Sharpe JA Parry DA Torelli S Childs AM , and others. 2014. Loss-of-function mutations in MICU1 cause a brain and muscle disorder linked to primary alterations in mitochondrial calcium signaling. Nat Genet 46:188–93. 10.1038/ng.2851 24336167

[bibr64-10738584211069060] Luca F Perry GH di Rienzo A. 2010. Evolutionary adaptations to dietary changes. Annu Rev Nutr 30:291–314. 20420525 10.1146/annurev-nutr-080508-141048 PMC4163920

[bibr65-10738584211069060] Lui JH Nowakowski TJ Pollen AA Javaherian A Kriegstein AR Oldham MC . 2014. Radial glia require PDGFD-PDGFRβ signalling in human but not mouse neocortex. Nature 515:264–8. 10.1038/nature13973 PMC4231536 25391964

[bibr66-10738584211069060] Lunt SY van der Heiden MG . 2011. Aerobic glycolysis: Meeting the metabolic requirements of cell proliferation. Annu Rev Cell Dev Biol 27:441–64. 10.1146/annurev-cellbio-092910-154237 21985671

[bibr67-10738584211069060] Mansour AA Gonçalves JT Bloyd CW Li H Fernandes S Quang D , and others. 2018. An in vivo model of functional and vascularized human brain organoids. Nat Biotechnol 36:432–41. 10.1038/nbt.4127 PMC6331203 29658944

[bibr68-10738584211069060] Marchi S Vitto VAM Patergnani S Pinton P . 2019. High mitochondrial Ca^2+^ content increases cancer cell proliferation upon inhibition of mitochondrial permeability transition pore (mPTP). Cell Cycle 18:914–6. 10.1080/15384101.2019.1598729 PMC6527303 30909805

[bibr69-10738584211069060] Maus A Peters GJ . 2017. Glutamate and α-ketoglutarate: key players in glioma metabolism. Amino Acids 49:21–32. 27752843 10.1007/s00726-016-2342-9 PMC5241329

[bibr70-10738584211069060] Meyer E Ricketts C Morgan NV Morris MR Pasha S Tee LJ , and others. 2010. Mutations in FLVCR2 are associated with proliferative vasculopathy and hydranencephaly-hydrocephaly syndrome (Fowler syndrome). Am J Hum Genet 86:471. 20206334 10.1016/j.ajhg.2010.02.004 PMC2833392

[bibr71-10738584211069060] Mirzaa G Dodge NN Glass I Day C Gripp K Nicholson L , and others. 2004. Megalencephaly and perisylvian polymicrogyria with postaxial polydactyly and hydrocephalus: a rare brain malformation syndrome associated with mental retardation and seizures. Neuropediatrics 35:353–9. 10.1055/s-2004-830497 15627943

[bibr72-10738584211069060] Molinari F Kaminska A Fiermonte G Boddaert N Raas-Rothschild A Plouin P , and others. 2009. Mutations in the mitochondrial glutamate carrier SLC25A22 in neonatal epileptic encephalopathy with suppression bursts. Clin Genet 76:188–94. 10.1111/j.1399-0004.2009.01236.x 19780765

[bibr73-10738584211069060] Mossmann D Park S Hall MN . 2018. MTOR signalling and cellular metabolism are mutual determinants in cancer. Nat Rev Cancer 18:744–57. 10.1038/s41568-018-0074-8 30425336

[bibr74-10738584211069060] Najmabadi H Hu H Garshasbi M Zemojtel T Abedini SS Chen W , and others. 2011. Deep sequencing reveals 50 novel genes for recessive cognitive disorders. Nature 478:57–63. 21937992 10.1038/nature10423

[bibr75-10738584211069060] Namba T Dóczi J Pinson A Xing L Kalebic N Wilsch-Bräuninger M , and others. 2020. Human-specific ARHGAP11B acts in mitochondria to expand neocortical progenitors by glutaminolysis. Neuron 105:867–881.e9. 31883789 10.1016/j.neuron.2019.11.027

[bibr76-10738584211069060] Namba T Huttner WB . 2017. Neural progenitor cells and their role in the development and evolutionary expansion of the neocortex. Wiley Interdiscip Rev Dev Biol 6(1). 10.1002/wdev.256 27865053

[bibr77-10738584211069060] Namba T Nardelli J Gressens P Huttner WB . 2021. Metabolic regulation of neocortical expansion in development and evolution. Neuron 109:408–19. 10.1016/j.neuron.2020.11.014 33306962

[bibr78-10738584211069060] Nashimoto Y Hayashi T Kunita I Nakamasu A Torisawa Y Nakayama M , and others. 2017. Integrating perfusable vascular networks with a three-dimensional tissue in a microfluidic device. Integr Biol 9:506–18. 10.1039/c7ib00024c 28561127

[bibr79-10738584211069060] Nowakowski TJ Bhaduri A Pollen AA Alvarado B Mostajo-Radji MA di Lullo E , and others. 2017. Spatiotemporal gene expression trajectories reveal developmental hierarchies of the human cortex. Science 358:1318–23. 10.1126/science.aap8809 PMC5991609 29217575

[bibr80-10738584211069060] Parks SK Mueller-Klieser W Pouysségur J . 2020. Lactate and acidity in the cancer microenvironment. Annu Rev Cancer Biol 4:141–58.

[bibr81-10738584211069060] Pinson A Namba T Huttner WB . 2019. Malformations of human neocortex in development: their progenitor cell basis and experimental model systems. Front Cell Neurosci 13:305. 31338027 10.3389/fncel.2019.00305 PMC6629864

[bibr82-10738584211069060] Pirozzi CJ Yan H. 2021. The implications of IDH mutations for cancer development and therapy. Nat Rev Clin Oncol 18:645–61. 10.1038/s41571-021-00521-0 34131315

[bibr83-10738584211069060] Poduri A Evrony GD Cai X Elhosary PC Beroukhim R Lehtinen MK , and others. 2012. Somatic activation of AKT3 causes hemispheric developmental brain malformations. Neuron 74:41–8. 10.1016/j.neuron.2012.03.010 PMC3460551 22500628

[bibr84-10738584211069060] Pollen AA Bhaduri A Andrews MG Nowakowski TJ Meyerson OS Mostajo-Radji MA , and others. 2019. Establishing cerebral organoids as models of human-specific brain evolution. Cell 176:743–756.e17. 30735633 10.1016/j.cell.2019.01.017 PMC6544371

[bibr85-10738584211069060] Qian X Su Y Adam CD Deutschmann AU Pather SR Goldberg EM , and others. 2020. Sliced human cortical organoids for modeling distinct cortical layer formation. Cell Stem Cell 26:766–781.e9. 32142682 10.1016/j.stem.2020.02.002 PMC7366517

[bibr86-10738584211069060] Rakic P . 2009. Evolution of the neocortex: a perspective from developmental biology. Nat Rev Neurosci 10:724–35. 10.1038/nrn2719 PMC2913577 19763105

[bibr87-10738584211069060] Rash BG Duque A Morozov YM Arellano JI Micali N Rakic P . 2019. Gliogenesis in the outer subventricular zone promotes enlargement and gyrification of the primate cerebrum. Proc Natl Acad Sci U S A 116:7089–94. 10.1073/pnas.1822169116 PMC6452694 30894491

[bibr88-10738584211069060] Rash BG Micali N Huttner AJ Morozov YM Horvath TL Rakic P . 2018. Metabolic regulation and glucose sensitivity of cortical radial glial cells. Proc Natl Acad Sci U S A 115:10142–7. 10.1073/pnas.1808066115 PMC6176632 30224493

[bibr89-10738584211069060] Rosenberg MJ Agarwala R Bouffard G Davis J Fiermonte G Hilliard MS , and others. 2002. Mutant deoxynucleotide carrier is associated with congenital microcephaly. Nat Genet 32:175–9. 10.1038/ng948 12185364

[bibr90-10738584211069060] Ruzzo EK Capo-Chichi JM Ben-Zeev B Chitayat D Mao H Pappas AL , and others. 2013. Deficiency of asparagine synthetase causes congenital microcephaly and a progressive form of encephalopathy. Neuron 80:429–41. 10.1016/j.neuron.2013.08.013 PMC3820368 24139043

[bibr91-10738584211069060] Shi Y Sun L Wang M Liu J Zhong S Li R , and others. 2020. Vascularized human cortical organoids (vOrganoids) model cortical development in vivo. PLoS Biol 18:e3000705. 32401820 10.1371/journal.pbio.3000705 PMC7250475

[bibr92-10738584211069060] Sies H Jones DP . 2020. Reactive oxygen species (ROS) as pleiotropic physiological signalling agents. Nat Rev Mol Cell Biol 21:363–83. 10.1038/s41580-020-0230-3 32231263

[bibr93-10738584211069060] Smart IHM, Dehay C, Giroud P, Berland M, Kennedy H. 2002. Unique morphological features of the proliferative zones and postmitotic compartments of the neural epithelium giving rise to striate and extrastriate cortex in the monkey. Cereb Cortex 12:37–53. 11734531 10.1093/cercor/12.1.37 PMC1931430

[bibr94-10738584211069060] Stepien BK Naumann R Holtz A Helppi J Huttner WB Vaid S. 2020. Lengthening neurogenic period during neocortical development causes a hallmark of neocortex expansion. Curr Biol 30:4227–4237.e5. 32888487 10.1016/j.cub.2020.08.046

[bibr95-10738584211069060] Sun T Hevner RF . 2014. Growth and folding of the mammalian cerebral cortex: from molecules to malformations. Nat Rev Neurosci 15:217–32. 10.1038/nrn3707 PMC4107216 24646670

[bibr96-10738584211069060] Suzuki IK Gacquer D van Heurck R Kumar D Wojno M Bilheu A , and others. 2018. Human-specific NOTCH2NL genes expand cortical neurogenesis through delta/notch regulation. Cell 173:1370–1384.e16. 29856955 10.1016/j.cell.2018.03.067 PMC6092419

[bibr97-10738584211069060] Szabadkai G Bianchi K Várnai P de Stefani D Wieckowski MR Cavagna D , and others. 2006. Chaperone-mediated coupling of endoplasmic reticulum and mitochondrial Ca^2+^ channels. J Cell Biol 175:901–11. 10.1083/jcb.200608073 PMC2064700 17178908

[bibr98-10738584211069060] TeSlaa T Chaikovsky AC Lipchina I Escobar SL Hochedlinger K Huang J , and others. 2016. α-Ketoglutarate accelerates the initial differentiation of primed human pluripotent stem cells. Cell Metab 24:485–93. 10.1016/j.cmet.2016.07.002 PMC5023506 27476976

[bibr99-10738584211069060] Thornton GK Woods CG . 2009. Primary microcephaly: do all roads lead to Rome? Trends Genet 25:501–10. 10.1016/j.tig.2009.09.011 PMC2816178 19850369

[bibr100-10738584211069060] Vaid S Camp JG Hersemann L Oegema CE Heninger AK Winkler S , and others. 2018. A novel population of hopx-dependent basal radial glial cells in the developing mouse neocortex. Development (Cambridge) 145:dev169276. 10.1242/dev.169276 30266827

[bibr101-10738584211069060] van den Ameele J Tiberi L Vanderhaeghen P Espuny-Camacho I . 2014. Thinking out of the dish: what to learn about cortical development using pluripotent stem cells. Trends Neurosci 37:334–42. 10.1016/j.tins.2014.03.005 24745669

[bibr102-10738584211069060] Vanhaesebroeck B Stephens L Hawkins P . 2012. PI3K signalling: the path to discovery and understanding. Nat Rev Mol Cell Biol 13:195–203. 22358332 10.1038/nrm3290

[bibr103-10738584211069060] Verloes A Drunat S Gressens P Passemard S. 1993. Primary autosomal recessive microcephalies and Seckel syndrome spectrum disorders. GeneReviews. 20301772

[bibr104-10738584211069060] Williams GSB Boyman L Lederer WJ . 2015. Mitochondrial calcium and the regulation of metabolism in the heart. J Mol Cell Cardiol 78:35–45. 25450609 10.1016/j.yjmcc.2014.10.019 PMC6534814

[bibr105-10738584211069060] Wishart DS Lewis MJ Morrissey JA Flegel MD Jeroncic K Xiong Y , and others. 2008. The human cerebrospinal fluid metabolome. J Chromatogr B 871:164–73. 10.1016/j.jchromb.2008.05.001 18502700

[bibr106-10738584211069060] Xie Z Jones A Deeney JT Hur SK Bankaitis VA . 2016. Inborn errors of long-chain fatty acid β-oxidation link neural stem cell self-renewal to autism. Cell Rep 14:991–9. 10.1016/j.celrep.2016.01.004 PMC4749429 26832401

[bibr107-10738584211069060] Xing L Kalebic N Namba T Vaid S Wimberger P Huttner WB . 2020. Serotonin receptor 2A activation promotes evolutionarily relevant basal progenitor proliferation in the developing neocortex. Neuron 108:1113–1129.e6. 33080227 10.1016/j.neuron.2020.09.034

[bibr108-10738584211069060] Xu IMJ Lai RKH Lin SH Tse APW Chiu DKC Koh HY , and others. 2016. Transketolase counteracts oxidative stress to drive cancer development. Proc Natl Acad Sci U S A 113:E725–E734. 26811478 10.1073/pnas.1508779113 PMC4760787

[bibr109-10738584211069060] Yang L Li Z Liu G Li X Yang Z. 2021. Developmental origins of human cortical oligodendrocytes and astrocytes. Neurosci Bull. Epub Aug 10. 10.1007/s12264-021-00759-9 PMC8783027 34374948

[bibr110-10738584211069060] Yang L Venneti S Nagrath D . 2017. Glutaminolysis: a hallmark of cancer metabolism. Annu Rev Biomed Eng 19:163–94. 10.1146/annurev-bioeng-071516-044546 28301735

[bibr111-10738584211069060] Zhang W Ma L Yang M Shao Q Xu J Lu Z , and others. 2020. Cerebral organoid and mouse models reveal a RAB39b-PI3K-mTOR pathway-dependent dysregulation of cortical development leading to macrocephaly/autism phenotypes. Genes Dev 34:580–97. 10.1101/gad.332494.119 PMC7111266 32115408

[bibr112-10738584211069060] Zhao Y Pan X Zhao J Wang Y Peng Y Zhong C . 2009. Decreased transketolase activity contributes to impaired hippocampal neurogenesis induced by thiamine deficiency. J Neurochem 111:537–46. 10.1111/j.1471-4159.2009.06341.x 19686241

[bibr113-10738584211069060] Zheng X Boyer L Jin M Mertens J Kim Y Ma L , and others. 2016. Metabolic reprogramming during neuronal differentiation from aerobic glycolysis to neuronal oxidative phosphorylation. eLife 5:e13374. 27282387 10.7554/eLife.13374 PMC4963198

[bibr114-10738584211069060] Zilles K Palomero-Gallagher N Amunts K. 2013. Development of cortical folding during evolution and ontogeny. Trends Neurosci 36:275–84. 10.1016/j.tins.2013.01.006 23415112

